# Supplementary material for: Estimating the impact of trained midwives and upgraded health facilities on institutional delivery rates in Nigeria using a quasi-experimental study design
Source: BMJ Open. 2022 May 19;12(5):e053792. doi: 10.1136/bmjopen-2021-053792 (PMC9125714; doi:10.1136/bmjopen-2021-053792)
Supplement: Supplementary data [file bmjopen-2021-053792supp001.pdf]

Supplementary Materials

The Wald test of the common trend assumption between 2008 and 2012 was never rejected for our main outcomes: institutional delivery (P-value = 0.30) and attendance of at least four antenatal visits during delivery (P-value = 0.24). Figures 1 and 2 show the trends, with confidence intervals, for these two outcome variables in the intervention and comparison groups during the 4-year pre-programme period; they confirm that pre-programme trends are roughly parallel when accounting for sampling noise. The common trend test is, however, rejected for infant mortality (p-value=0.016) and skilled birth attendance (p-value=0.046), hence we refrain from reporting results for these outcomes.

Figure 1: More than four time of ANC visits during pregnancy (catchment area=2500m, buffer=100m)

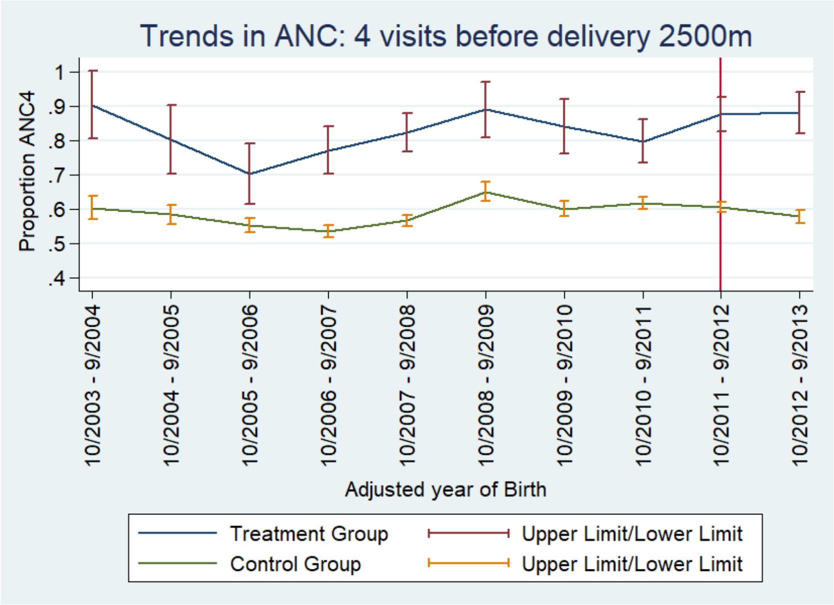

Figure 2: Skilled delivery (Catchment area=2500m, buffer=100m)

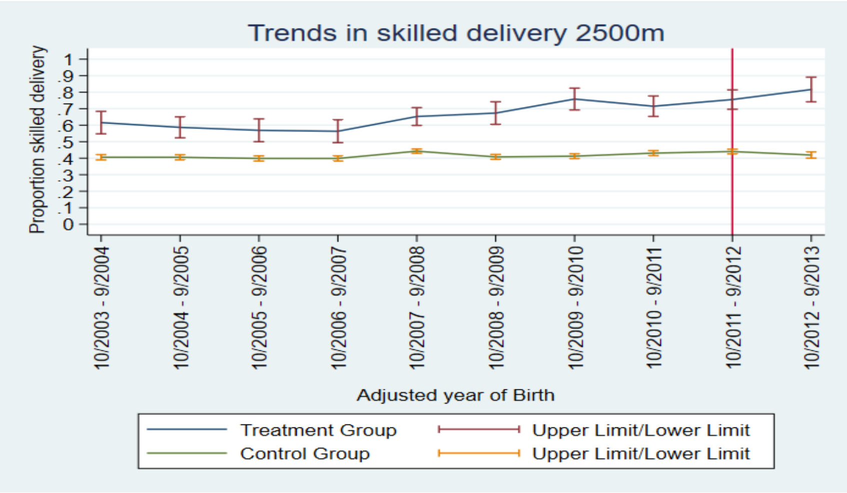

Figure 3: Institutional delivery (Catchment area=2500m, buffer=100m)

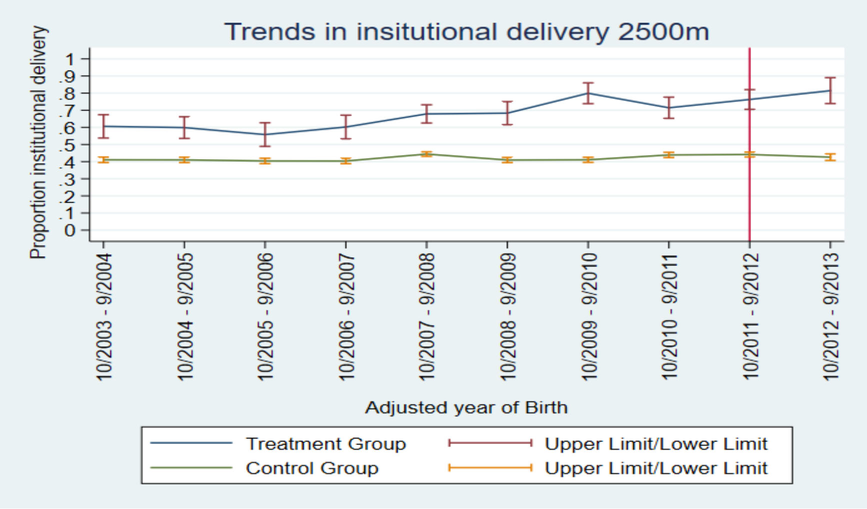

Table 1: Robustness check, estimates including data from the 2018 NDHS

First year of SURE-P MCH : Oct 2012 - Oct 2013 (with controls)

	**DID Coef**	95% Confidence Interval		P-value
Institutional delivery				
2000m	**0.079**	0.00	0.15	**0.04**
2500m	**0.068**	0.00	0.14	**0.06**
3000m	**0.045**	-0.01	0.10	**0.14**
At least 4 times of ANC visits				
2000m	**0.031**	-0.03	0.10	**0.35**
2500m	**0.032**	-0.02	0.09	**0.26**
3000m	**0.002**	-0.06	0.06	**0.95**

Second (final) year of SURE-P MCH: Oct 2013 - Oct 2014 (with controls)

	**DID Coef**	95% Confidence Interval		P-value
Institutional delivery				
2000m	**-0.015**	-0.14	0.02	**0.16**
2500m	**0.019**	-0.10	0.07	**0.67**
3000m	**-0.015**	-0.09	0.06	**0.68**
At least 4 times of ANC visits				
2000m	**-0.078**	-0.19	0.04	**0.18**
2500m	**0.052**	-0.15	0.04	**0.29**
3000m	**-0.049**	-0.13	0.04	**0.26**
