# Supplementary material for: Estimating the impact of trained midwives and upgraded health facilities on institutional delivery rates in Nigeria using a quasi-experimental study design
Source: BMJ Open. 2022 May 19;12(5):e053792. doi: 10.1136/bmjopen-2021-053792 (PMC9125714; doi:10.1136/bmjopen-2021-053792)
Supplement: Author's manuscript [file bmjopen-2021-053792.draft_revisions.pdf]

BMJ Open

BMJ Open is committed to open peer review. As part of this commitment we make the peer review history of every article we publish publicly available.

When an article is published we post the peer reviewers' comments and the authors' responses online. We also post the versions of the paper that were used during peer review. These are the versions that the peer review comments apply to.

The versions of the paper that follow are the versions that were submitted during the peer review process. They are not the versions of record or the final published versions. They should not be cited or distributed as the published version of this manuscript.

BMJ Open is an open access journal and the full, final, typeset and author-corrected version of record of the manuscript is available on our site with no access controls, subscription charges or pay-per-view fees (http://bmjopen.bmj.com).

If you have any questions on BMJ Open's open peer review process please email info.bmjopen@bmj.com

# The impact of trained midwives and upgraded health facilities on institutional delivery rates in Nigeria



| | |
|---|---|
| Journal: | *BMJ Open* |
| Manuscript ID | bmjopen-2021-053792 |
| Article Type: | Original research |
| Date Submitted by the Author: | 01-Jun-2021 |
| Complete List of Authors: | Grépin, Karen; University of Hong Kong Li Ka Shing Faculty of Medicine, School of Public Health<br>Chukwuma, Adanna ; World Bank, Development Research Group<br>Vera-Hernandez, Marcos; University College London, Economics<br>Wang, Qiao; World Bank, Development Research Group<br>Rosa-Dias, Pedro; Imperial College London, Associate Professor of Health Economics |
| Keywords: | Health economics < HEALTH SERVICES ADMINISTRATION & MANAGEMENT, Health policy < HEALTH SERVICES ADMINISTRATION & MANAGEMENT, International health services < HEALTH SERVICES ADMINISTRATION & MANAGEMENT, Maternal medicine < OBSTETRICS, PUBLIC HEALTH |
| | |

BMJ

*I, the Submitting Author has the right to grant and does grant on behalf of all authors of the Work (as defined in the below author licence), an exclusive licence and/or a non-exclusive licence for contributions from authors who are: i) UK Crown employees; ii) where BMJ has agreed a CC-BY licence shall apply, and/or iii) in accordance with the terms applicable for US Federal Government officers or employees acting as part of their official duties; on a worldwide, perpetual, irrevocable, royalty-free basis to BMJ Publishing Group Ltd ("BMJ") its licensees and where the relevant Journal is co-owned by BMJ to the co-owners of the Journal, to publish the Work in this journal and any other BMJ products and to exploit all rights, as set out in our licence.*

*The Submitting Author accepts and understands that any supply made under these terms is made by BMJ to the Submitting Author unless you are acting as an employee on behalf of your employer or a postgraduate student of an affiliated institution which is paying any applicable article publishing charge ("APC") for Open Access articles. Where the Submitting Author wishes to make the Work available on an Open Access basis (and intends to pay the relevant APC), the terms of reuse of such Open Access shall be governed by a Creative Commons licence – details of these licences and which Creative Commons licence will apply to this Work are set out in our licence referred to above.*

*Other than as permitted in any relevant BMJ Author's Self Archiving Policies, I confirm this Work has not been accepted for publication elsewhere, is not being considered for publication elsewhere and does not duplicate material already published. I confirm all authors consent to publication of this Work and authorise the granting of this licence.*

For peer review only

**Title**: The impact of trained midwives and upgraded health facilities on institutional delivery rates in Nigeria

**Authors:**

Karen Ann Grépin[1]

University of Hong, Kong Li Ka Shing Faculty of Medicine, School of Public Health
7 Sassoon Road, Hong Kong SAR

Adanna Chukwuma

World Bank, Development Research Group, Washington, DC, USA

Marcos Vera-Hernandez

University College London, Economics, London, UK

Qiao Wang

World Bank, Development Research Group, Washington, DC, USA

Pedro Rosa-Dias

Imperial College London, Associate Professor of Health Economics, London, UK

**Keywords**: health economics, health policy, international health services, maternal health, public health

**Word Count**: 3290

[1] Corresponding author: School of Public Health, 7 Sassoon Road, Pokfulam, Hong Kong SAR; kgrepin@hku.hk, +852 3917 9719

For peer review only

## Abstract:

**Introduction**: Maternal and neonatal mortality rates are negatively associated with the proportion of women giving birth in a health facility across in low and middle-income countries. Studies have shown that demand-side interventions, such as conditional cash transfers and vouchers, can increase the use of health services, but evidence of the effectiveness of supply-side interventions, including those aimed at improving the availability of skilled birth attendants in health facilities, is limited in these settings.

**Methods**: We evaluated the impact of the Subsidy Reinvestment and Empowerment Programme Maternal and Child Health Project (SURE-P), a large-scale programme that allocated trained midwives and upgraded health facilities in under resourced areas across Nigeria, using a differences-in-differences (DID) study design that compared changes in rates of institutional delivery and antenatal care rates in areas that received additional support through the programme relative to areas that did not. Data on outcomes were obtained from the 2013 Nigerian Demographic and Health Survey.

**Results**: We found that the programme significantly increased the proportion of women giving birth in a health facility by approximately 7 percentage points, or approximately 10% relative to the baseline, after 9 months of implementation but not on the use of antenatal care.

**Conclusion**: The findings of this study suggest there could be important improvements in institutional delivery rates through greater investment in supply-side interventions.


**Funding**: Bill & Melinda Gates Foundation and the Strategic Impact Evaluation Fund



For peer review only - http://bmjopen.bmj.com/site/about/guidelines.xhtml

## Article Summary

### Strengths and limitations of the study

- This project evaluates the impact of a real-world study that was implemented at scale across Nigeria on the use of maternal health services.
- Our study combined programmatic data with household survey data to estimate the impact of the program at the individual level using data from the Nigerian Demographic and Health Survey.
- Our study finds robust evidence that the scale-up of a trained midwife program, coupled with facilities upgrades, led to important increases in the rates of institutional deliveries in Nigeria.
- Due to the reliance on household survey data that was not collected for the purposes of this study, we were limited by sample size and the types of outcomes we could evaluate.
- A lack of precise geographic information on household may have limited our ability to attribute effects of the program directly.

For peer review only

## Introduction

The world has made great progress in reducing maternal mortality: the estimated number of women dying in childbirth declined from 385 per 100,000 live births in 1990s to 216 in 2015.[1] Global progress, however, has not been even and countries in Sub-Saharan Africa (SSA) have made the least progress. Nigeria alone accounted for almost a fifth of the estimated maternal deaths that occurred globally in 2015 and was estimated to have one of the highest maternal mortality ratios in the world at 814 per 100,000 live births.[2]

Over the same time period, the proportion of women giving birth in a health facility has substantially increased in SSA.[3] However, the lack of commensurate improvements in maternal health outcomes in some countries suggests that the indicators used to monitor maternal health progress may not be fully capturing the elements of the content or of the quality of care during childbirth that are essential to improve health outcomes.[4] Many of the root causes of maternal mortality have not changed in decades: far too many women are accessing health services "too little, too late".[5] And although more women are giving birth in a health facility, and in the presence of a SBA, if those health facilities are not adequately equipped, or if the SBA is not adequately trained and empowered to provide high quality care, health outcomes may not improve.

While there is a clear need to improve the quality of maternal health care in such settings,[6] there is little rigorous evidence of the impact of such investments on the use of health services. In particular, and although studies have shown that many demand side interventions (e.g. conditional cash transfers and vouchers)[7] can greatly improve the use of health services, there is less robust evidence of the effectiveness of supply side interventions, including those aimed at improving the availability of SBAs and the quality of healthcare facilities.[8,9] Beyond

For peer review only

ensuring the presence of a SBA, many factors also limit their ability to deliver high quality health services, including a lack of training and supervision, excessive workloads, low salaries and poor living conditions, as well as a lack of access to well-equipped health facilities. The impact of making improvements in these other dimensions is largely not well understood.[10]

Countries in SSA, including Nigeria, rely upon a range of cadres of health professionals to provide specialized care to woman, infants, and families over the entire continuum of pregnancy from pre-conception to the early weeks of life, all of which are classified as SBAs.[11] Midwives and nurses are among the most common types of health professionals that provide skilled assistance at delivery across SSA.[12,13] Historically, important reductions in maternal mortality have been linked to the expansion of midwives in high income countries, for example in Sweden.[14] Less is known about the protentional contribution of expanded access to midwives in low and middle-income countries (LMICs) today. A recent modelling study, however, suggests that the widespread scale-up of midwifery in LMICs could avert a substantial proportion of reproductive-related morbidity and mortality, although rigorous evidence of scaling up midwives and other support has not been established.[15]

Building off this gap in the literature, this study aims to evaluate the impact of the Subsidy Reinvestment and Empowerment Programme Maternal and Child Health Project (SURE-P), a large-scale programme launched in 2012 to accelerate progress towards the Millennium Development Goals (MDGs) 4 and 5 in Nigeria, on institutional delivery and antenatal care rates. The goal is to generate rigorous evidence on the potential effectiveness of similar large scale- programmes to improve coverage of high-quality health services in other high maternal mortality settings.

For peer review only

## Methods

### *Study Context*

In 2012, the Federal Government of Nigeria launched SURE-P using reduced subsidies on petroleum consumption to fund the programme. The programme was implemented by the National Primary Health Care Development Agency, a parastatal organization located under the Federal Ministry of Health. The programme recruited, trained, and deployed midwives to public primary health care facilities (PHCs) in high-priority areas across Nigeria's 36 States and the Federal Capital Territory. Facilities were purposively selected for the project based on need, which was defined as a persistent lack of midwives, and conditional on meeting minimum infrastructure and human resource requirements. The programme also provided upgrades to eligible PHCs and provided supplies of drugs to facilitate midwives' work in these areas. To be eligible for SURE-P, PHCs needed to have running water, electricity for at least some hours, and a room to store medicines and equipment. Some PHCs that didn't meet these conditions but could be inexpensively upgraded to meet them also received basic infrastructure upgrades to qualify for the programme. Between September 2012 and May 2013, the programme deployed 1285 midwives to 473 PHCs. Although there had not initially been a planned end-date for the programme, it was cut short after less than a year of implementation due to a budget shortfall related to a sharp fall in international oil prices.

### *Evaluation Strategy*

To evaluate the impact of SURE-P, we adopted a differences-in-differences (DID) study design, a method that has been widely used to rigorously evaluate the impact of health and social programmes in other contexts.[16] The approach compares the change in outcomes in treated areas to the change in outcomes in comparison areas and attributes any difference in changes to the programme. We define exposure to the programme at the individual-level, specifically

For peer review only



For peer review only - http://bmjopen.bmj.com/site/about/guidelines.xhtml

we define women who live in areas where SURE-P was implemented to be exposed, which we operationalize with a binary treatment variable. We also define a post-treatment variable, which takes value 1 if a birth occurs after the programme was implemented, or 0 otherwise. For the purposes of this analysis, we consider October 1, 2012 the date in which the programme started to be implemented, based on information obtained from programme officials. The effect of the programme is then estimated as the interaction of the treatment variable and the post-treatment variable, conditional on a set of covariates. We also account for sample clustering effects in our estimates of confidence intervals and p-values.

The DID approach relies upon a common trend assumption (i.e., parallel trends in the outcome indicators across treatment and comparison groups, in the absence of treatment), however, this assumption can never be formally tested in the presence of an intervention, so it is standard to test this assumption in the pre-treatment period. In the Results section we provide estimates of our test of the common trend assumption and find that a reasonable assumption of common trend holds for the key outcomes of our study.

***Data Sources***

Our source of data for outcome and control variables, which were measured at both the individual and household levels, was the 2013 Nigerian Demographic and Health Survey (NDHS),[17] which was conducted between February and June 2013. Data on the location of health facilities was obtained from the National MDGs Information System (NMIS),[18] which provides geo-referenced locations of over 34,000 health facilities.

To define the treatment and comparison status of health facilities we determined the location of all the 473 SURE-P facilities using programme data. We then determined a 100-meter radius



For peer review only - http://bmjopen.bmj.com/site/about/guidelines.xhtml

around each SURE-P facility and defined all facilities within such radius as treatment facilities, under the assumption that all facilities within the same proximity of a treatment facility were also indirectly affected by the programme. This produced a final list of treatment facilities in the NMIS, and the remaining facilities were defined as comparison facilities. Figure 1 provides a graphical representation of treatment and comparison facilities.

In the NDHS, households were sampled within primary sampling units (also called clusters), which were geo-coded. To determine whether each cluster from the 2013 NDHS was within a treatment or comparison area, we first determined the distance of each cluster to the nearest treated facility as well as the nearest comparison facility (in meters, using the geo-coordinates of the center of the cluster). We defined treatment clusters those located less than 2,500 meters from the nearest treated facility and defined comparison clusters as those located within the 2500 meters radius of a comparison facility and outside 2500 meters circle of a treatment facility. To avoid comparing households living in very remote locations with those located close to a treatment facility, we dropped from the analysis a small number of clusters that were located more 2,500 meters from the nearest treatment facility and over 7,500 meters from the nearest comparison facility. These distances were selected based on a review of the literature on distance-based access to care measures across Sub-Saharan Africa, accounting for the mean distance of clusters from facilities within the sample.[19] Nonetheless, in our Online Appendix, we perform robustness analysis by changing these thresholds extensively; our conclusions do not depend on the precise distance thresholds chosen.[20]

The two primary outcomes of the study were the rate of institutional deliveries (ID), which was defined as the proportion of deliveries as reported by women that took place in either a government hospital, health center, health post or other public sector medical facility, or a



For peer review only - http://bmjopen.bmj.com/site/about/guidelines.xhtml

private hospital, clinic, or other medical sector facility; and the percentage of all pregnancies resulting in a live birth for which the mother reported receiving at least four antenatal care visits are reported (ANC4).

A rich set of covariates was also drawn from the DHS dataset, including data collected at the individual, household, cluster, and regional levels. Covariates were selected based on whether they could have influenced facility selection criteria and thus represent a potential confounder with the primary outcome variables of interest. A summary of covariates is given in Table 1.

***Study registration and changes to protocol***

This study had been registered as an observational study.[21] At the time of registration, it was believed that we could collect a purposely designed survey in control areas to collect outcome variables. However, in the end, this was not possible due to lack of funding. As such, we ended up using the 2013 NDHS as a source of data for this evaluation. Among the secondary outcomes that we had registered, we could not analyze post-partum depression or pregnancy and obstetric-related health care practices because they were not available in the 2013 NDHS. This study obtained ethical approval, as part of a larger research project, from two separate institutional review boards: the University College London Research Ethics Committee (IRB approval number 1827/004, 2013/02/13) and the National Health Research Ethics Committee of Nigeria (approval number NHREC 01012007, 2013/02/02).

***Patient and public involvement statement***

Patients or the public were not involved in the design, or conduct, or reporting, or dissemination plans of our research.



For peer review only - http://bmjopen.bmj.com/site/about/guidelines.xhtml

## Results

Table 1 describes the characteristics of mothers and households in both the treatment and comparisons areas before the start of the intervention. Institutional delivery rates were much higher in the treatment than in comparison areas (72% vs. 43%), as was the percentage of births in which the mother had at least four antenatal care visits (85% vs. 61%). Additional characteristics (such as education and household income) confirm that the treatment areas were significantly better off than the comparison areas. However, it should be noted that the difference-in-difference method that we employ controls for differences in time-invariant unobservable variables that might affect the outcome of interest if the common trend assumption holds.

Table 2 shows the estimated programme effects on institutional delivery and attendance of at least four antenatal visits 9 months after programme implementation. For households within the 2,500-metre catchment area of a treatment facility, the results in panel 2 show that the programme increased institutional delivery rates by 6.7 percentage points and is statistically significant (p-value = 0.069). Without adjusting for covariates (panel 1), the effect is slightly larger (7.2 percentage points), but the confidence interval is wider. Hence, the covariates improve the precision of the estimates. Regarding the outcome of at least four antenatal visits, although the point estimates are positive, they are not statistically significant.

The main estimates were obtained assuming a facility catchment area of 2,500m (as suggested in Okwaraji and Edmond, 2012)[22] and using a radius of 100m to match SURE-P facilities between the NMIS database and the purposely collected database of SURE-P facilities. For robustness, in Exhibit 3, we also provide results for catchment areas of 2000m and 3000m. The results are very similar to the main estimates of 2500m, but slightly larger (7.4 percentage

For peer review only

points vs. 6.7 percentage points) and with a smaller p-value (0.051 vs. 0.069) for the catchment area of 2,000m compared to 2,500m. Assuming a catchment area of 3,000m compared to 2,500 these estimates are marginally smaller (5.0 percentage points vs. 6.7 percentage points) and with a larger p-value (0.09 vs. 0.069). Also, for robustness, we estimate the model using a facility-matching radius of 200 meters instead of 100 meters and found nearly identical results.

**Discussion**

Improving the quality of maternal health services is an important global health priority for many countries, including Nigeria. In 2012, it launched an ambitious programme that dispatched trained midwives to eligible health facilities across the country. This study found that the increased availability of the midwives led to substantial increases in the proportion of women giving birth in a health facility, leading to an increase in institutional delivery rates by 7.2 percentage points. This represents approximately a 10 percent proportional increase in women getting access to health services, a substantial increase obtained after nine months of implementation. This effect is however smaller than estimates found for demand-side interventions in SSA, including recent evidence on conditional cash transfers in Nigeria[7,23]. In addition, the increased availability of midwives did not cause an increase in the use of antenatal care while conditional cash transfer programme have been shown to be effective in increasing antenatal care use in other settings,[7,23] which is an important mechanism for subsequent improvements in maternal and neo-natal health outcomes.

From a policy perspective the evidence in this study indicates that supply-side interventions that increase the availability of SBAs can have substantial effects even in the short-run, but also that the magnitude of these effects and the range of health outcomes can likely be improved if these are complemented by suitable demand-side initiatives, such as conditional cash


For peer review only - http://bmjopen.bmj.com/site/about/guidelines.xhtml

transfers. More research is needed on the complementarity between demand and supply-side policies in this context, as well as on the role of the quality of the services provided.

Our study has a few limitations that must be taken into consideration in the interpretation of our results. First, the DHS data were collected in clusters which were geo-referenced, but this locational data is displaced to protect the identity of respondents[24]. This means that we may have incorrectly classified some treatment clusters as comparison clusters, and vice-versa. However, with this type of measurement error, we would have been more likely to have misclassified treatment clusters as controls, which should have bias downwards our estimates of impact as the SURE-P programme, not the other way. Second, the NMIS database was our only source of geographic information on non-SURE-P facilities and these data were collected in 2012, a year before our outcomes, which could have led to some discrepancies in location as a result. Third, due to our reliance on the NDHS for our key outcomes, our unit of analysis was a birth, not a pregnancy. In other words, we have data on all births, but not necessarily on all pregnancies. It is possible that differential outcomes between the treatment and comparison groups were also affected by differential pregnancy termination rates. However, we do not find a significant difference in the proportion of women who had ever terminated a pregnancy between treatment and control areas after the programme started. Fifth, we cannot rule out the possibility that women in DHS clusters classified as being in the comparison group, responded to the SURE-P programme by seeking care in treatment facilities. However, the procedure used to assign treatment or comparison group status to each DHS cluster means that all control clusters were at least 2,500 meters from treatment facilities. Based on a review of the literature on distance-based access to care measures across Sub-Saharan Africa, we therefore expect access of the control group to treatment facilities to be limited.[21] Also, our sensitivity analysis indicates that our results are robust to varying the threshold. Finally, the extent to which the



For peer review only - http://bmjopen.bmj.com/site/about/guidelines.xhtml

results found here could be replicated in a broader set of Nigerian primary healthcare facilities is uncertain. Although SURE-P was implemented in all Nigerian states and the Federal Capital Territory, the facilities selected for SURE-P were, on average, better off in terms of our main outcomes. This was because treatment facilities were partly selected based on the availability of human resources and equipment.

## Conclusion

The MDGs focused a great deal of attention and resources to improving maternal health outcomes globally, and while the MDGs for maternal and child health were not met in Nigeria, the results of this study demonstrate that supply side improvements could hold promise to further increase rates of institutional delivery in LMICs. Supply side interventions, which thus far have been relatively less investigated, thus represent an under investigated part of the solution to maternal mortality. More research is needed to understand the impact of other supply side improvements, including the complimentary role they can plan alongside demand side incentives, on health outcomes in Nigeria and in other international contexts.

## Contributions of authors

- KAG was involved in the analysis and writing of the original and final manuscripts.
- AC was involved in conceptualizing this project, developing the methodology, administering this project, obtaining funding, and reviewing and editing the manuscript.
- MV-H was involved in conceptualizing this project, developing the methodology, administering this project, obtaining funding, managing project resources, and reviewing and editing the manuscript.
- MH was involved in conceptualizing this project, developing the methodology, obtaining funding for the project, administering the project, and managing project resources.

For peer review only

- QW was involved in developing the methodology for the project, curating the data collected in the project, administering the project, and providing resources for the project.
- PRD was involved in the conceptualization of the research project, conducting the analysis for the paper, obtaining funding for the project, developing the research project methodology, administering the project, writing the original draft of the paper, and reviewing and editing the final version of the manuscript.

**Data sharing statement**

Programmatic data are available upon request by emailing PRD. The data on institutional delivers were obtained from publicly available data sources.

For peer review only

## Endnotes

1 Alkema L, Chou D, Hogan D, et al. Global, regional, and national levels and trends in maternal mortality between 1990 and 2015, with scenario-based projections to 2030: a systematic analysis by the UN Maternal Mortality Estimation Inter-Agency Group. The Lancet 2016; 387(10017): 462-74.

2 Bongaarts J. WHO, UNICEF, UNFPA, World Bank Group, and United Nations Population Division Trends in Maternal Mortality: 1990 to 2015 Geneva: World Health Organization, 2015. Population and Development Review 2016; 42(4): 726-.

3 Collaboration Ct, Boerma T, Requejo J, et al. Countdown to 2030: tracking progress towards universal coverage for reproductive, maternal, newborn, and child health. The Lancet 2018; 391(10129): 1538-48.

4 Campbell OMR, Calvert C, Testa A, et al. The scale, scope, coverage, and capability of childbirth care. The Lancet 2016; 388(0).

5 McDougall L, Campbell OMR, Graham W. Maternal Health: An Executive Summary for The Lancet's Series. The Lancet 2016.

6 Kruk ME, Leslie HH, Verguet S, Mbaruku GM, Adanu RMK, Langer A. Quality of basic maternal care functions in health facilities of five African countries: an analysis of national health system surveys. The Lancet Global Health 2016; 4(11): e845 e55.

7 Okeke, E. N., Wagner, Z., & Abubakar, I. S. Maternal cash transfers led to increases in facility deliveries and improved quality of delivery care in Nigeria. Health Affairs 2020; 39(6): 1051-1059.

8 Grépin KA, Habyarimana J, Jack W. Cash on delivery: Results of a randomized experiment to promote maternal health care in Kenya. Journal of Health Economics 2019; 65(Economic Development and Cultural Change 2016): 15-30.

9 De Walque D, Friedman J, Neelsen S, Wagstaff A. Systematic Review and Meta-Analysis of Impacts of Supply-and Demand-Side Financial Incentives in Health in Low-and Middle-Income Countries. 2019: iHEA.

10 Munabi-Babigumira S, Glenton C, Lewin S, Fretheim A, Nabudere H. Factors that influence the provision of intrapartum and postnatal care by skilled birth attendants in low- and middle-income countries: a qualitative evidence synthesis. Cochrane Database of Systematic Reviews 2017; 11(11): CD011558.

11 Hoope-Bender Pt, Bernis Ld, Campbell J, et al. Improvement of maternal and newborn health through midwifery. The Lancet 2014; 384(9949): 1226-35.

12 Lerberghe WV, Matthews Z, Achadi E, et al. Country experience with strengthening of health systems and deployment of midwives in countries with high maternal mortality. The Lancet 2014; 384(9949): 1215-25.



For peer review only - http://bmjopen.bmj.com/site/about/guidelines.xhtml

13 Adegoke A, Utz B, Msuya SE, Broek Nvd. Skilled Birth Attendants: Who is Who? A Descriptive Study of Definitions and Roles from Nine Sub Saharan African Countries. PLoS ONE 2012; 7(7): e40220.

14 Högberg U. The Decline in Maternal Mortality in Sweden: The Role of Community Midwifery. American Journal of Public Health 2004; 94(8): 1312-20.

15 Homer CSE, Friberg IK, Dias MAB, et al. The projected effect of scaling up midwifery. The Lancet 2014; 384(9948): 1146-57.

16 Heckman J, Robb R. Alternative methods for evaluating the impact of interventions: An overview. Journal of Econometrics 1985; 30(1-2): 239-67.

17 National Population Commission - NPC/Nigeria, ICF International. Nigeria Demographic and Health Survey 2013. Abuja, Nigeria: NPC/Nigeria and ICF International, 2014.

18 About NMIS. Nigeria MDG Information System. https://myf.github.io/nmis/about (accessed Feb 11, 2021).

19 Okwaraji YB, Edmond KM. Proximity to health services and child survival in low- and middle-income countries: a systematic review and meta-analysis. BMJ Open 2012; 2(4): e001196.

20. To access the Appendix, click on the Appendix link in the box to the right of the article online

21 ISRCTN. Improving maternal and child health care in Nigeria: an assessment of the maternal and child care component of the Subsidy Reinvestment & Empowerment Programme (SURE-p MCH). http://www.who.int/trialsearch/Trial2aspx?TrialID=ISRCTN98056858 2013; (1): 1-8.

22 Okwaraji YB, Edmond KM. Proximity to health services and child survival in low- and middle-income countries: a systematic review and meta-analysis. BMJ Open 2012; 2(4): e001196.

23 Okeke, E. N., & Abubakar, I. S. Healthcare at the beginning of life and child survival: Evidence from a cash transfer experiment in Nigeria. Journal of Development Economics 2020: 143:102426.

24 Burgert CR, Colston J, Roy T, Zachary B. Geographic displacement procedure and georeferenced data release policy for the Demographic and Health Surveys [Internet]. Calverton, Maryland, USA: ICF International; 2013. (DHS Spatial Analysis Reports No. 7). Available from: http://dhsprogram.com/pubs/pdf/SAR7/SAR7.pdf

For peer review only

Figure 1: Matching NMIS facilities and SURE-P facilities

For peer review only


For peer review only - http://bmjopen.bmj.com/site/about/guidelines.xhtml

Table 1: Summary of outcomes, characteristics of mother and household by study groups before the intervention

| | Total | | Intervention | | Comparison | | Difference | |
|---|---|---|---|---|---|---|---|---|
| Outcomes | N | % | N | % | N | % | Perc. Pt | p-value |
| Delivery assistance: doctor/nurse/midwife | 19599 | 0.43 | 891 | 0.70 | 18708 | 0.42 | 0.305 | 0.000 |
| Delivery in any facility | 19671 | 0.44 | 891 | 0.72 | 18780 | 0.43 | 0.317 | 0.000 |
| >=4 times of antenatal visits during pregnancy | 11533 | 0.62 | 511 | 0.85 | 11022 | 0.61 | 0.244 | 0.000 |
| | Total | | Intervention | | Comparison | | Difference | |
| Covariates | N | % | N | % | N | % | Perc. Pt | P-value |
| maternal age | 21755 | 27.62 | 975 | 28.01 | 20780 | 27.60 | 0.748 | 0.106 |
| parity at delivery | 21755 | 2.84 | 975 | 2.24 | 20780 | 2.87 | -0.613 | 0.000 |
| covered by health insurance | 14074 | 0.02 | 648 | 0.03 | 13426 | 0.02 | 0.004 | 0.592 |
| currently working | 14065 | 0.73 | 645 | 0.80 | 13420 | 0.73 | 0.094 | 0.000 |
| husband/partner has at least primary school education | 13594 | 0.71 | 613 | 0.90 | 12981 | 0.70 | 0.206 | 0.000 |
| respondent has at least primary school education | 14136 | 0.63 | 648 | 0.86 | 13488 | 0.62 | 0.266 | 0.000 |
| currently married or in union | 14136 | 0.93 | 648 | 0.91 | 13488 | 0.93 | -0.025 | 0.026 |
| respondent is Muslim | 14075 | 0.53 | 646 | 0.35 | 13429 | 0.54 | -0.264 | 0.003 |
| respondent reads newspapers | 14136 | 0.18 | 648 | 0.29 | 13488 | 0.18 | 0.104 | 0.014 |
| respondent listens to radio | 14136 | 0.66 | 648 | 0.75 | 13488 | 0.65 | 0.078 | 0.042 |
| respondent watches TV | 14136 | 0.55 | 648 | 0.77 | 13488 | 0.54 | 0.229 | 0.001 |
| household has electricity | 12519 | 0.59 | 608 | 0.81 | 11911 | 0.58 | 0.268 | 0.000 |
| belongs to the poorest two quintiles | 12634 | 0.32 | 613 | 0.09 | 12021 | 0.33 | -0.241 | 0.000 |
| residence is urban | 12634 | 0.47 | 613 | 0.71 | 12021 | 0.45 | 0.285 | 0.001 |
| residence in North East | 12634 | 0.15 | 613 | 0.05 | 12021 | 0.15 | -0.097 | 0.000 |
| residence in North West | 12634 | 0.26 | 613 | 0.05 | 12021 | 0.27 | -0.260 | 0.000 |
| residence in South East | 12634 | 0.11 | 613 | 0.22 | 12021 | 0.10 | 0.190 | 0.107 |
| residence in South South | 12634 | 0.15 | 613 | 0.15 | 12021 | 0.15 | 0.035 | 0.631 |
| residence in South West | 12634 | 0.17 | 613 | 0.36 | 12021 | 0.16 | 0.171 | 0.078 |

note: (1) buffer=100m, and catchment area=2500m
(2) for birth record level variables, baseline is defined as births that were prior to October 2012 when SURE-P MCH programme started; (3) for respondent women level and household level variables, with using DHS 2013 only, the statistics are "follow-up", as those respondent women and households that were interviewed in 2013



For peer review only - http://bmjopen.bmj.com/site/about/guidelines.xhtml

Table 2: OLS regressions – effect of the intervention on institutional deliveries and use of antenatal care

Panel 1: Unadjusted (no controls)

| | DID Coef | 95% Confidence Interval | | | | P-value | N |
|---|---|---|---|---|---|---|---|
| Institutional delivery | | | | | | | |
| 2000m | 0.075 | [ | -0.042 | 0.193 | ] | 0.209 | 19475 |
| 2500m | 0.072 | [ | -0.032 | 0.175 | ] | 0.175 | 22343 |
| 3000m | 0.050 | [ | -0.047 | 0.146 | ] | 0.316 | 24524 |
| At least 4 times of ANC visits | | | | | | | |
| 2000m | 0.048 | [ | -0.034 | 0.131 | ] | 0.254 | 12279 |
| 2500m | 0.059 | [ | -0.012 | 0.130 | ] | 0.103 | 14095 |
| 3000m | 0.032 | [ | -0.044 | 0.109 | ] | 0.406 | 15473 |

Panel 2: Adjusted (with controls)

| | DID Coef | 95% Confidence Interval | | | | P-value | N |
|---|---|---|---|---|---|---|---|
| Institutional delivery | | | | | | | |
| 2000m | 0.074 | [ | -0.000 | 0.148 | ] | 0.051 | 18413 |
| 2500m | 0.067 | [ | -0.005 | 0.138 | ] | 0.069 | 21130 |
| 3000m | 0.050 | [ | -0.008 | 0.107 | ] | 0.090 | 23240 |
| At least 4 times of ANC visits | | | | | | | |
| 2000m | 0.029 | [ | -0.037 | 0.095 | ] | 0.393 | 11488 |
| 2500m | 0.030 | [ | -0.028 | 0.087 | ] | 0.311 | 13200 |
| 3000m | 0.020 | [ | -0.029 | 0.069 | ] | 0.420 | 14526 |

notes:

(1) Control variables include both mother's characteristics (maternal age, square of maternal age, birth order, mother's health insurance coverage, current working status, mother and husband/partner's education level, mother's marriage status, religion, and exposure to media) and household characteristics (access to electricity and asset quintiles).

(2) All standard errors are robust and clustered at DHS cluster level.



For peer review only - http://bmjopen.bmj.com/site/about/guidelines.xhtml

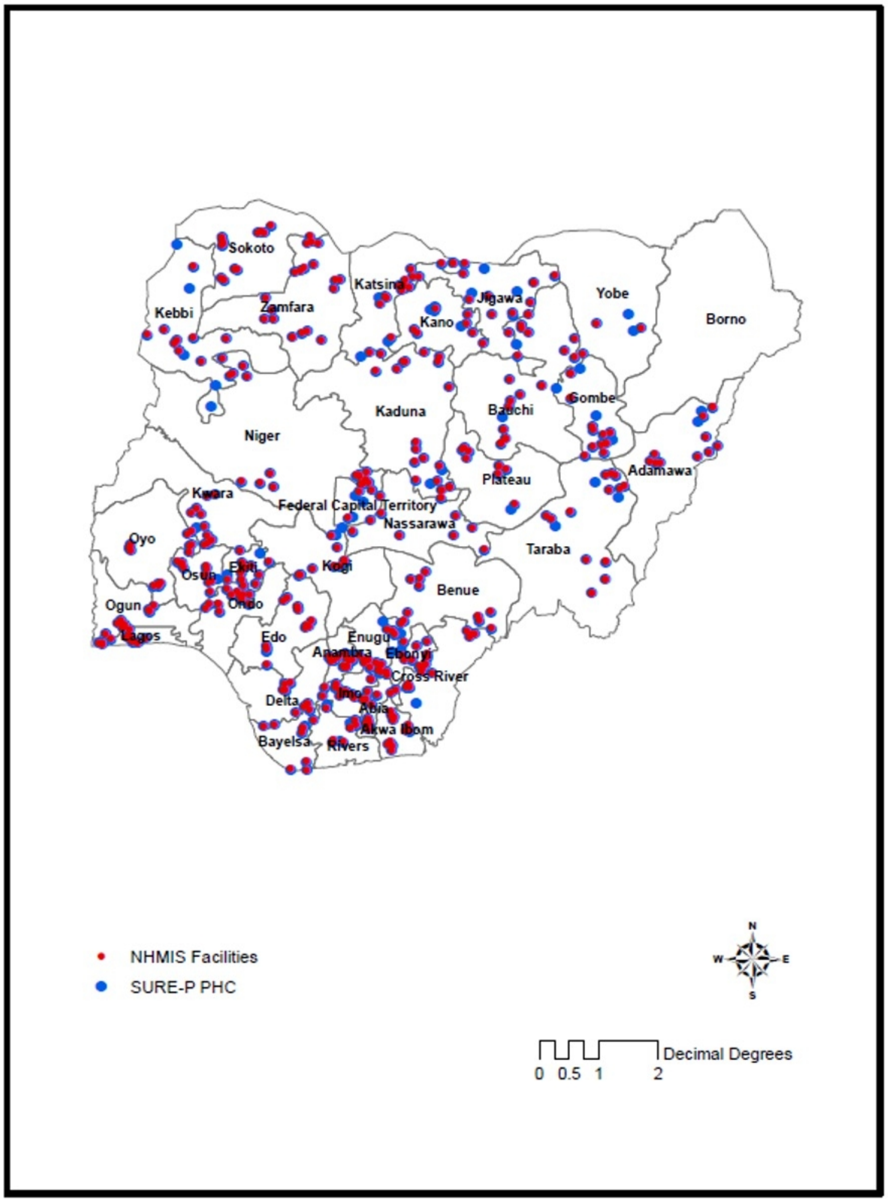


Figure 1: Matching NMIS facilities and SURE-P facilities

155x209mm (144 x 144 DPI)

## Supplementary Materials

The Wald test of the common trend assumption between 2008 and 2012 was never rejected for our main outcomes: institutional delivery (P-value = 0.30) and attendance of at least four antenatal visits during delivery (P-value = 0.24). Figures 1 and 2 show the trends, with confidence intervals, for these two outcome variables in the intervention and comparison groups during the 4-year pre-programme period; they confirm that pre-programme trends are roughly parallel when accounting for sampling noise. The common trend test is, however, rejected for infant mortality (p-value=0.016) and skilled birth attendance (p-value=0.046), hence we refrain from reporting results for these outcomes.

Figure 1: More than four time of ANC visits during pregnancy (catchment area=2500m, buffer=100m)

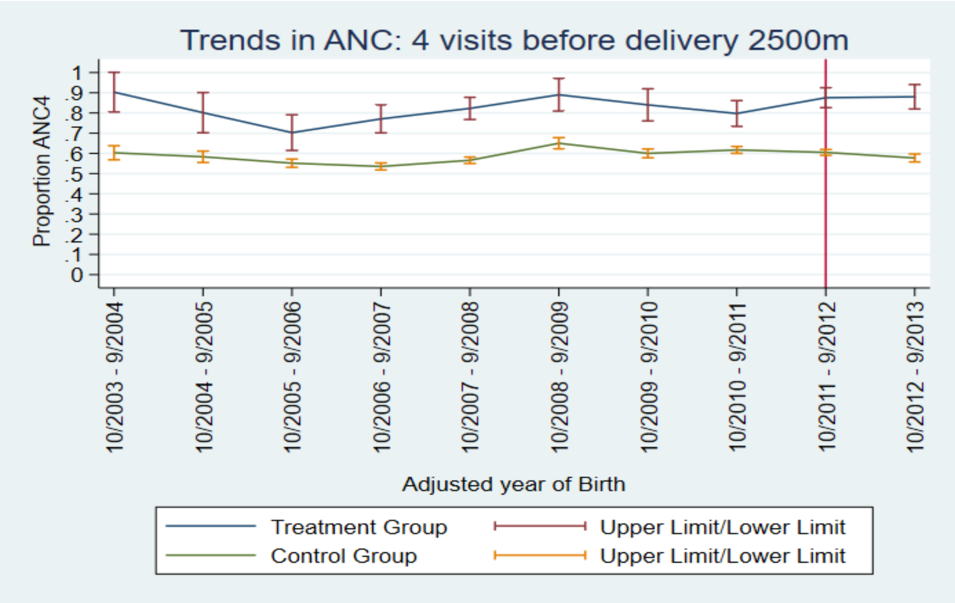


Figure 2: Skilled delivery (Catchment area=2500m, buffer=100m)

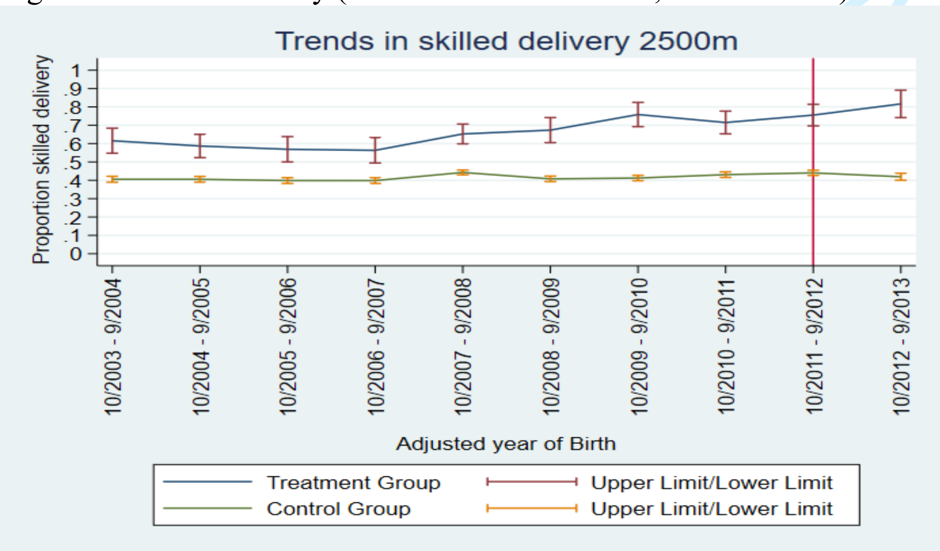


For peer review only - http://bmjopen.bmj.com/site/about/guidelines.xhtml

Figure 3: Institutional delivery (Catchment area=2500m, buffer=100m)

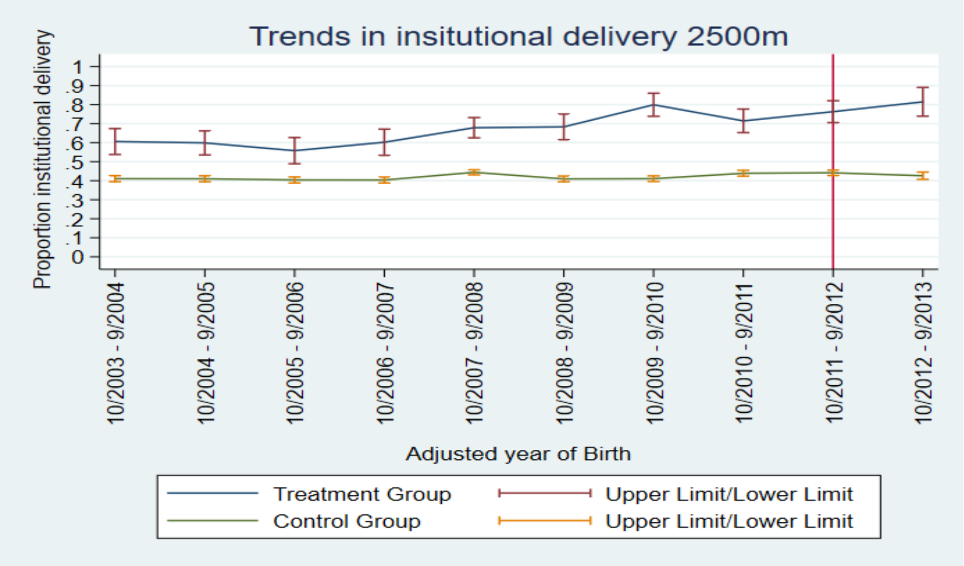


For peer review only

BMJ Open

# Estimating the impact of trained midwives and upgraded health facilities on institutional delivery rates in Nigeria using a quasi-experimental study design

| | |
|---|---|
| Journal: | *BMJ Open* |
| Manuscript ID | bmjopen-2021-053792.R1 |
| Article Type: | Original research |
| Date Submitted by the Author: | 11-Mar-2022 |
| Complete List of Authors: | Grépin, Karen; University of Hong Kong Li Ka Shing Faculty of Medicine, School of Public Health<br>Chukwuma, Adanna ; World Bank, Development Research Group<br>Vera-Hernandez, Marcos; University College London, Economics<br>Wang, Qiao; World Bank, Development Research Group<br>Rosa-Dias, Pedro; Imperial College London, Associate Professor of Health Economics |
| <b>Primary Subject Heading</b>: | Health economics |
| Secondary Subject Heading: | Global health, Health policy |
| Keywords: | Health economics < HEALTH SERVICES ADMINISTRATION & MANAGEMENT, Health policy < HEALTH SERVICES ADMINISTRATION & MANAGEMENT, International health services < HEALTH SERVICES ADMINISTRATION & MANAGEMENT, Maternal medicine < OBSTETRICS, PUBLIC HEALTH |
| | |

SCHOLARONE™
Manuscripts

*I, the Submitting Author has the right to grant and does grant on behalf of all authors of the Work (as defined in the below author licence), an exclusive licence and/or a non-exclusive licence for contributions from authors who are: i) UK Crown employees; ii) where BMJ has agreed a CC-BY licence shall apply, and/or iii) in accordance with the terms applicable for US Federal Government officers or employees acting as part of their official duties; on a worldwide, perpetual, irrevocable, royalty-free basis to BMJ Publishing Group Ltd ("BMJ") its licensees and where the relevant Journal is co-owned by BMJ to the co-owners of the Journal, to publish the Work in this journal and any other BMJ products and to exploit all rights, as set out in our licence.*

*The Submitting Author accepts and understands that any supply made under these terms is made by BMJ to the Submitting Author unless you are acting as an employee on behalf of your employer or a postgraduate student of an affiliated institution which is paying any applicable article publishing charge ("APC") for Open Access articles. Where the Submitting Author wishes to make the Work available on an Open Access basis (and intends to pay the relevant APC), the terms of reuse of such Open Access shall be governed by a Creative Commons licence – details of these licences and which Creative Commons licence will apply to this Work are set out in our licence referred to above.*

*Other than as permitted in any relevant BMJ Author's Self Archiving Policies, I confirm this Work has not been accepted for publication elsewhere, is not being considered for publication elsewhere and does not duplicate material already published. I confirm all authors consent to publication of this Work and authorise the granting of this licence.*

For peer review only

**Title**: Estimating the impact of trained midwives and upgraded health facilities on institutional delivery rates in Nigeria using a quasi-experimental study design

**Authors:**

Karen Ann Grépin

University of Hong, Kong Li Ka Shing Faculty of Medicine, School of Public Health
7 Sassoon Road, Hong Kong SAR

Adanna Chukwuma

World Bank, Development Research Group, Washington, DC, USA

Marcus Holmlund

World Bank, Development Impact Evaluation Group, Washington, DC, USA

Marcos Vera-Hernandez

University College London, Economics, London, UK

Qiao Wang

World Bank, Development Research Group, Washington, DC, USA

Pedro Rosa-Dias[1]

Imperial College London, Associate Professor of Health Economics, London, UK

**Keywords**: health economics, health policy, international health services, maternal health, public health

**Word Count**: 3290

[1] Corresponding author: Business School, Imperial College London, South Kensington Campus, London, UK SW7 2AZ, +44 (0)20 7589 5111



For peer review only - http://bmjopen.bmj.com/site/about/guidelines.xhtml

**Abstract**:

**Objectives**: Studies have shown that demand-side interventions, such as conditional cash transfers and vouchers, can increase the proportion of women giving birth in a health facility in low and middle-income countries, but there is limited evidence of the effectiveness of supply-side interventions. We evaluated the impact of the Subsidy Reinvestment and Empowerment Programme Maternal and Child Health Project (SURE-P MCH) on rates of institutional delivery and antenatal care.

**Design, setting, and participants**: We used a differences-in-differences study design that compared changes in rates of institutional delivery and antenatal care rates in areas that had received additional support through the SURE-P MCH programme relative to areas that did not. Data on outcomes were obtained from the 2013 Nigerian Demographic and Health Survey.

**Results**: We found that the programme significantly increased the proportion of women giving birth in a health facility by approximately 7 percentage points (p-value 0.069) or approximately 10% relative to the baseline after 9 months of implementation. The programme, however, did not significantly increase the use of antenatal care.

**Conclusion**: The findings of this study suggest there could be important improvements in institutional delivery rates through greater investment in supply-side interventions.

**Funding**: Bill & Melinda Gates Foundation and the Strategic Impact Evaluation Fund

For peer review only

## Article Summary

### Strengths and limitations of the study

- This project evaluated the impact of a real-world programme that was implemented at scale on the use of maternal health services across Nigeria.
- The programme provided trained midwives and facility upgrades to participating primary health centres.
- Our study combined programmatic data with household survey data to estimate the impact of the programme at the individual level using data from the Nigerian Demographic and Health Survey.
- Due to the reliance on household survey data that were not collected for the purposes of this study, we were limited by sample size and the types of outcomes we could evaluate.
- A lack of precise geographic information on households may have limited our ability to directly attribute effects of the programme.

For peer review only

## Introduction

The world has made great advancements in reducing maternal mortality: the estimated number of women dying in childbirth declined from 385 per 100,000 live births in 1990s to 216 in 2015.[1] Progress, however, has not been even and countries in Sub-Saharan Africa (SSA) have made the least improvements. Nigeria alone accounted for almost a fifth of the estimated maternal deaths that occurred globally in 2015 and was estimated to have one of the highest maternal mortality ratios in the world at 814 per 100,000 live births.[2]

Over the same time period, the proportion of women giving birth in a health facility has also substantially increased in SSA.[3] However, the lack of commensurate improvements in maternal health outcomes in some countries suggests that the indicators used to monitor maternal health progress may not be fully capturing the elements of the content or of the quality of care received during childbirth that are essential to improve health outcomes.[4] Many of the root causes of maternal mortality have not changed in decades: far too many women are accessing health services "too little, too late".[5] And although more women are giving birth in a health facility, and in the presence of a skilled birth attendant (SBA), if those health facilities are not adequately equipped, or if the SBA is not adequately trained and empowered to provide high quality care, then health outcomes may not improve.

While there is a clear need to improve the quality of maternal health care in such settings,[6] there is little rigorous evidence of the impact of such investments on the use of health services. And although studies have shown that many demand side interventions (e.g. conditional cash transfers and vouchers)[7] can greatly improve the use of health services, there is less rigorous evidence of the effectiveness of supply side interventions, including those aimed at improving the availability of SBAs and the quality of healthcare facilities.[8,9] Beyond ensuring the

For peer review only

presence of a SBA, many factors also limit the ability of SBAs to deliver high quality health services, including lack of training and supervision, excessive workloads, low salaries and poor living conditions, as well as lack of access to well-equipped health facilities. The impact of making improvements in these other dimensions is for the most part not well understood.[10]

Countries in SSA, including Nigeria, rely upon a range of cadres of health professionals to provide specialized care to woman, infants, and families over the entire continuum of pregnancy from pre-conception to the early weeks of life and all of these health professionals are classified as SBAs.[11] Midwives and nurses are among the most common types of health professionals that provide skilled assistance across SSA.[12,13] Historically, important reductions in maternal mortality have been linked to the expansion of midwives in high income countries like Sweden.[14] But less is known about the potential contribution of greater access to midwives in low and middle-income countries (LMICs) today. However, a recent modelling study found that the scale-up of midwifery in LMICs could avert a substantial proportion of reproductive-related morbidity and mortality in these countries, although rigorous evidence of scaling up midwives and other related support has not been established.[15]

Building off this gap in the literature, this study aims to evaluate the impact of the Subsidy Reinvestment and Empowerment Programme Maternal and Child Health Project (SURE-P MCH), a large-scale programme introduced in 2012 that deployed trained midwives and was aimed at accelerating progress towards Millennium Development Goals (MDGs) 4 and 5, on institutional delivery and antenatal care rates across Nigeria. Our goal with this study is to generate rigorous evidence on the potential effectiveness of comparable large scale-programmes to improve coverage of high-quality health services in other high maternal mortality settings.

For peer review only - http://bmjopen.bmj.com/site/about/guidelines.xhtml

## Methods

### *Study Context*

In January 2012, the Federal Government of Nigeria eliminated a longstanding fuel subsidy and announced that funds raised by the removal of this subsidy would be used to improve public services, including health services. Following this announcement, the SURE-P MCH programme was launched as a flagship initiative to improve maternal and child health outcomes across the country. The programme was implemented by the National Primary Health Care Development Agency, a parastatal organization under the Federal Ministry of Health.

Following its launch, the programme began the process of upgrading primary health centres (PHCs) training of newly recruited midwives. In order to facilitate the rapid roll-out of the programme , facilities were purposively selected for the project based on need (defined as a persistent lack of midwives) and was also conditional on meeting a set of minimum infrastructure and human resource requirements. To be eligible for SURE-P MCH, PHCs had to: offer maternal and child health services; have minimum equipment and basic infrastructure, including potable water supply, power supply, and sewage disposal; and operate on a twenty-four-hour basis. These criteria, especially the last condition, explain why our treated facilities and their respective catchment areas, had higher rates of institutional delivery at baseline than our comparison facilities.

Following their training, the programme deployed 1,285 midwives to 473 PHCs in high-priority areas across Nigeria's 36 States and the Federal Capital Territory. Each facility usually received more than one additional midwife and the first batch of facilities began to fully participate in the program in October 2012. The SURE-P MCH programme also benefited

For peer review only

from a wide-reaching mass media campaign, encompassing radio and television adverts, billboards, and posters encouraging pregnant women to visit SURE-P MCH PHRCs after the deployment of the midwives.

Although initially there had been plans to further scale up the programme after the initial phase, unfortunately the programme had been cut short about a year after implementation primarily due a budget shortfall related to a sharp fall in international oil prices. Although the programme was not officially terminated until 2015, news had begun to circulate following the drop in oil prices that the programme would be terminated in early 2014. Programme officials had reported to the research team high levels of midwife attrition, which may have started in late 2013. Due to these factors, we limit our analysis to the first year of programme implementation (October 2012-2013), however, as discussed in the data section, we only had data for the first 9 months of this implementation period to evaluate outcomes.

***Evaluation Strategy***

To evaluate the impact of SURE-P MCH, we adopted a differences-in-differences (DID) study design, a method that has been widely used to rigorously evaluate the impact of health and social programmes in other comparable contexts.[16] The approach compares the change in outcomes in treated areas to the change in outcomes in comparison areas, attributing any difference in changes to the programme. We define exposure to the programme at the individual-level, more specifically we define women who live in areas where SURE-P MCH was implemented to be exposed, which we operationalize with a binary treatment variable. We also define a post-treatment variable, which takes the value of 1 if a birth occurs after the programme was implemented, or 0 otherwise.



For peer review only - http://bmjopen.bmj.com/site/about/guidelines.xhtml

For the purposes of this analysis, we consider October 1, 2012 as the start date of the programme, based on information obtained from national programme officials. The effect of the programme is then estimated as the interaction of the treatment variable and the post-treatment variable, conditional on a set of covariates. Our regression model is thus the one below, where X is a vector of covariates listed in Table 1:

$$Y_{it} = \alpha_{it} + \beta_1 SUREp_{it} + \beta_2 Post_t + \beta_3 SUREp_{it} * Post_t + \Sigma X_{it} + \varepsilon_{it}$$

In this equation, Y are the outcome variables of interest (institutional deliveries or antenatal care), SURE is a binary variable if the mother lives in a household that is located in one of the SURE-P MCH treatment areas or not, and Post is a dummy variable that takes the value of 1 if the birth occurred after October 2012. X is a set of control variables, which are fully described in our result tables. We also account for sample clustering effects in our estimates of confidence intervals and p-values.

The DID approach relies upon a common trend assumption (i.e. parallel trends in the outcome indicators across treatment and comparison groups, in the absence of treatment), however, this assumption can never be formally tested in the presence of an intervention, thus it is standard to test this assumption in the pre-treatment period. In the Results Section we provide estimates of our test of the common trend assumption and show that the parallel trends hypothesis cannot be rejected for the entire pre-treatment period.

***Data Sources***

Our data source for the outcome and control variables, which were measured at both the individual and household levels, was the 2013 Nigerian Demographic and Health Survey (NDHS)[17] conducted between February and June 2013. Furthermore, the data on the location



For peer review only - http://bmjopen.bmj.com/site/about/guidelines.xhtml

of health facilities obtained from the National MDGs Information System (NMIS),[18] provides geo-referenced locations of over 34,000 health facilities.

In order to define the treatment and comparison status of health facilities, we ascertained the location of all the 473 SURE-P MCH facilities using programme data. We then determined a 100-meter radius around each SURE-P MCH facility and defined all facilities within such radius as treatment facilities, under the assumption that all facilities within the same proximity of a treatment facility were indirectly affected by the programme. This produced a final list of treatment facilities in the NMIS, and the remaining facilities were later defined as comparison facilities.

In the NDHS, households were sampled within primary sampling units (also called clusters), and these were geo-coded. To determine whether each cluster from the 2013 NDHS was within a treatment or comparison area, we first found the distance of each cluster to the nearest treatment facility as well as the nearest comparison facility (in metres, using the geo-coordinates of the center of the cluster). We defined treatment clusters as those located less than 2,500 metres from the nearest treatment facility and comparison clusters as those located within a 2,500-metre radius of a comparison facility and outside a 2,500-metres radius of a treatment facility. To avoid comparing households living in very remote locations with those located close to a treatment facility, our analysis excluded a small number of clusters that were located more 2,500 metres from the nearest treatment facility and over 7,500 metres from the nearest comparison facility. The selected distances were derived from a literature review on distance-based access to care measures across Sub-Saharan Africa that accounted for the mean distance of clusters from facilities within the sample.[19] Nonetheless, in our Online Appendix,



For peer review only - http://bmjopen.bmj.com/site/about/guidelines.xhtml

we performed a robustness analysis by changing these thresholds; ultimately our conclusions do not depend on the precise distance of the chosen thresholds chosen.[20]

The two primary outcomes of the study were the rate of institutional deliveries (ID), defined as the proportion of deliveries, as reported by women, that took place in either a government hospital, health center, health post or other public sector medical facility, a private hospital, clinic or other medical sector facility; and the percentage of all pregnancies resulting in a live birth for which the mother reported receiving at least four antenatal care visits (ANC4).

Given that our treatment areas differ from the comparison areas due to the selection criteria of the facilities, a rich set of covariates was also drawn from the DHS dataset including data collected at the individual, household, cluster, and regional levels. The covariates were selected based on whether they could have influenced the facility selection criteria and thus represent a potential confounder with the primary outcome variables of interest. A summary of covariates is given in Table 1.

***Study registration and changes to protocol***

This study was originally registered as an observational study.[21] At the time of registration, it was hoped that we could collect a purposely designed survey in the comparison areas to collect the outcome variables. However, in the end, this was not possible due to a lack of funding. As such, we used the 2013 NDHS as our data source for this evaluation. Among the secondary outcomes that we had previously registered, we could not analyse post-partum depression or pregnancy and obstetric-related health care practices because they could not be obtained from the 2013 NDHS. This study obtained ethical approval, as part of a larger research project, from two separate institutional review boards: the University College London Research Ethics

For peer review only

Committee (IRB approval number 1827/004, 2013/02/13) and the National Health Research Ethics Committee of Nigeria (approval number NHREC 01012007, 2013/02/02).

***Patient and public involvement statement***

Patients or the public were not involved in the design, conduct, reporting or dissemination plans of our research.

## Results

Table 1 describes the characteristics of mothers and households in both the treatment and comparisons areas before the start of the intervention. Institutional delivery rates were much higher in the treatment areas than in comparison areas (72% vs. 43%), as was the percentage of births in which the mother had at least four antenatal care visits (85% vs. 61%). Additional characteristics (such as education and household income) confirm that the treatment areas were significantly better off than the comparison areas. However, it should be noted that the difference-in-difference method that we employed controls for differences in time-invariant unobservable variables that might affect the outcome of interest if the common trend assumption holds.

Although it is not possible to statistically prove that trends in institutional deliveries were parallel prior to the start of the programme, we used hypothesis testing to ascertain whether our data are consistent with the hypothesis of parallel trends in the pre-policy period. Specifically, we performed a Wald test of the hypotheses of common trend assumption and these were never rejected during the pre-policy period years (2008 to 2012) for institutional deliveries (p-value = 0.30) and attendance of at least four antenatal visits during delivery (p-value = 0.24). This is also consistent with the assumption, which is true to the best of our knowledge, that there

For peer review only

were no other large-scale policies or programmes in place in Nigeria that would have affected the SURE-P MCH facilities differently than those in the comparison areas, which would be difficult given the very specific criteria that were used to select the treatment facilities. We previously discussed these criteria in our Study Context section.

Table 2 shows the estimated programme effects on institutional delivery and attendance of at least four antenatal visits 9 months after implementation of the programme. For households within the 2,500-metre catchment area of a treatment facility, the results in panel 2 show that the programme increased institutional delivery rates by 6.7 percentage points and is statistically significant (p-value = 0.069) at the 10% level. And without adjusting for covariates (panel 1), the effect is slightly larger (7.2 percentage points), but the confidence interval is wider. Hence, the covariates improve the precision of the estimates. As for the outcome of ANC4, although the point estimates are positive, they are not statistically significant.

The main estimates were obtained assuming a facility catchment area of 2,500 metres (as suggested in Okwaraji and Edmond, 2012)[22] and using a radius of 100 metres to match SURE-P MCH facilities between the NMIS database and the purposely collected database of SURE-P MCH facilities. For robustness, in Exhibit 3, we also provide results for catchment areas of 2,000 metres and 3,000 metres. The results are very similar to the main estimates of 2,500 metres, but slightly larger (7.4 percentage points vs. 6.7 percentage points) and with a smaller p-value (0.051 vs. 0.069) for the catchment area of 2,000 metres compared to 2,500 metres. Furthermore, assuming a catchment area of 3,000 metres compared to that of 2,500 metres, the estimates are marginally smaller (5.0 percentage points vs. 6.7 percentage points) and with a larger p-value (0.09 vs. 0.069). Also, for robustness, we estimated the model using a facility-matching radius of 200 metres instead of 100 metres and found nearly identical results.


For peer review only - http://bmjopen.bmj.com/site/about/guidelines.xhtml

As a robustness check, we have re-estimated our model for the period October 2012 to October 2013 using all the births recorded in the 2013 NDHS as well additional births from 2013 and 2014 that were captured in the 2018 NDHS. However, only a very small number of additional births (2.8% of the births in the 2018 NDHS) were recorded in 2013. When we again estimate the impact of the programme in the first year, the results are similar to those seen Table 2 (and presented in Table 1 of the online appendix). In addition, when we estimate the effects of the programme using a in the second year of implementation, that is for the October 2013 – October 2014 we found no statistically significant effects of the programme on institutional delivery, which is consistent with our other observations that midwife attrition as well as financial and logistical programs had already begun to plague the programme after the first year. We therefore prefer the estimates of the first year of implementation only using the 2013 Nigerian DHS dataset only.

**Discussion**

Improving the quality of maternal health services is an important global health priority for many countries, including Nigeria. In 2012, the Nigerian government launched an ambitious programme that dispatched trained midwives to eligible health facilities across the country. This study found that the increased availability of the midwives led to substantial increases in the proportion of women giving birth in a health facility leading to an increase in institutional delivery rates by 7.2 percentage points. This represents approximately a 10 percent proportional increase in women gaining access to health services, a substantial increase obtained after nine months of implementation. However, the increased availability of midwives did not cause an increase in the use of antenatal care.


For peer review only - http://bmjopen.bmj.com/site/about/guidelines.xhtml

To contextualise our findings, we compared our findings to those observed in other studies of other programmes aimed at increasing institutional delivery rates in similar contexts. Our findings are smaller in magnitude than those found in evaluations of conditional cash transfer programmes in Nigeria.[7,23] However, a recent systematic review of the impact of demand side programmes on institutional delivery rates in low income settings found that financial incentive programmes could increase institutional delivery rates on average by 5.3 percentage points, with conditional cash transfer programmes had on average larger effects.[24] Therefore our from Nigeria findings are comparable to those observed in many demand side programmes.

While there is limited evidence of the impact of supply side interventions, a notable exception is a recent study by Croke et al., which investigated the impact of national health facility construction programme on delivery rates in Ethiopia using similar data and study design. The authors find similar effect sizes: the construction of a new health facility led lead to a 7.2 percentage point increase in institutional delivery rates amongst treated facilities and that the effects were observed almost immediately after the facilities had been constructed.[25] Proportionally our results are smaller, due to higher baseline health service utilization rates, however, taken together our study and those findings suggest that supply side interventions, when properly implemented, can also translate into meaningful gains in institutional delivery rates. More research is needed on the complementarity between demand and supply-side policies in this context, as well as on the role of the quality of the services provided.

However, while evidence from this study indicates that supply-side interventions that increase the availability of midwives and upgrade health facilities can have substantial effects in the short-run, it also highlights that it can be challenging to maintain large-scale national-level programmes in many international contexts. More evidence is needed to support the

For peer review only

development of programmes aimed at the supply side and efforts to sustain these programmes over the long-run.

Our study has a few limitations that must be taken into consideration when interpretating our results. First, the DHS data were collected in clusters which were geo-referenced but this locational data was displaced to protect the identity of respondents.[26] This means that we may have incorrectly classified some treatment clusters as comparison clusters or vice-versa. However, with this type of measurement error, we would have been more likely to have misclassified treatment clusters as comparison clusters, which should have biased downwards our estimates of the impact of the SURE-P MCH programme and not the other way around. Second, the NMIS database was our only source of geographic information on non-SURE-P MCH facilities and these data were collected in 2012, a year before our outcomes, which could have led to some discrepancies in location as a result. Third, due to our reliance on the NDHS for our key outcomes, our unit of analysis was a birth, not a pregnancy. In other words, we have data on all births, but not necessarily on all pregnancies. It is possible that the differential outcomes between the treatment and comparison groups were also affected by the differential pregnancy termination rates. However, we did not find a significant difference in the proportion of women who had terminated a pregnancy between treatment and comparison areas after the programme started (not shown, but data are available upon request). Fifth, we cannot rule out the possibility that women in DHS clusters classified as being in the comparison group responded to the SURE-P MCH programme by seeking care in treatment facilities. Regardless, the procedures used to assign treatment or comparison group status to each DHS cluster means that all comparison clusters were at least 2,500 metres from treatment facilities. Based on a literature review on distance-based access to care measures across SSA, we therefore expect access of the comparison group to treatment facilities to be limited.[21] Also, our sensitivity



For peer review only - http://bmjopen.bmj.com/site/about/guidelines.xhtml

analysis indicates that our results are robust to varying the threshold. Finally, the extent to which the results found here can be replicated within the broader Nigerian primary health care system is uncertain. Although SURE-P MCH was implemented in all Nigerian states and the Federal Capital Territory, the facilities selected for SURE-P MCH were, on average, better off in terms of our main outcomes. This was because treatment facilities were partly selected based on the availability of human resources and equipment.

## Conclusion

As a result of the MDGs, additional resources were channelled towards and a lot of the focus was placed on improving maternal health outcomes globally. And while the MDGs for maternal and child health were not met in Nigeria, the results of this study demonstrate that supply side improvements holds (great) promise for increased rates of institutional delivery in Nigeria and likely other LMICs. Supply-side interventions, which thus far have been poorly studied, represent an under investigated part of the solution to maternal mortality. Therefore, additional research is needed to understand the impact of other supply-side improvements, including the complementary role they can play alongside demand-side incentives, on health outcomes in Nigeria and in other international contexts.

## Competing Interests

Between 2012 and 2014, MVH and PRD declaring receiving short-term consultancy fees from the World Bank to support this evaluation project. KAG, QW, and AC declare no competing interests.

For peer review only

**Contributions of authors**

- KAG was involved in the analysis and writing of the original and final manuscripts.
- AC was involved in the conceptualization of this project, developing the methodology, administering this project, obtaining funding, reviewing, and editing the manuscript.
- MV-H was involved in the conceptualization of this project, developing the methodology, administering this project, obtaining funding, managing project resources, reviewing, and editing the manuscript.
- MH was involved in the conceptualization of this project, developing the methodology, obtaining funding for the project, administering the project, and managing project resources.
- QW was involved in developing the methodology for the project, curating the data collected for the project, administering the project, and providing resources for the project.
- PRD was involved in the conceptualization of the research project, conducting the analysis for the paper, obtaining funding for the project, developing the research project methodology, administering the project, writing the original draft of the paper, reviewing, and editing the final version of the manuscript.
- The funding agency had no role in the drafting of this manuscript.

**Data sharing statement**

Programmatic data are available upon request by emailing PRD. The data on institutional delivers were obtained from publicly available data sources.



For peer review only - http://bmjopen.bmj.com/site/about/guidelines.xhtml

For peer review only

## Endnotes

1 Alkema L, Chou D, Hogan D, et al. Global, regional, and national levels and trends in maternal mortality between 1990 and 2015, with scenario-based projections to 2030: a systematic analysis by the UN Maternal Mortality Estimation Inter-Agency Group. The Lancet 2016; 387(10017): 462-74.

2 Bongaarts J. WHO, UNICEF, UNFPA, World Bank Group, and United Nations Population Division Trends in Maternal Mortality: 1990 to 2015 Geneva: World Health Organization, 2015. Population and Development Review 2016; 42(4): 726-.

3 Collaboration Ct, Boerma T, Requejo J, et al. Countdown to 2030: tracking progress towards universal coverage for reproductive, maternal, newborn, and child health. The Lancet 2018; 391(10129): 1538-48.

4 Campbell OMR, Calvert C, Testa A, et al. The scale, scope, coverage, and capability of childbirth care. The Lancet 2016; 388(0).

5 McDougall L, Campbell OMR, Graham W. Maternal Health: An Executive Summary for The Lancet's Series. The Lancet 2016.

6 Kruk ME, Leslie HH, Verguet S, Mbaruku GM, Adanu RMK, Langer A. Quality of basic maternal care functions in health facilities of five African countries: an analysis of national health system surveys. The Lancet Global Health 2016; 4(11): e845 e55.

7 Okeke, E. N., Wagner, Z., & Abubakar, I. S. Maternal cash transfers led to increases in facility deliveries and improved quality of delivery care in Nigeria. Health Affairs 2020; 39(6): 1051-1059.

8 Grépin KA, Habyarimana J, Jack W. Cash on delivery: Results of a randomized experiment to promote maternal health care in Kenya. Journal of Health Economics 2019; 65(Economic Development and Cultural Change 2016): 15-30.

9 De Walque D, Friedman J, Neelsen S, Wagstaff A. Systematic Review and Meta-Analysis of Impacts of Supply-and Demand-Side Financial Incentives in Health in Low-and Middle-Income Countries. 2019: iHEA.

10 Munabi-Babigumira S, Glenton C, Lewin S, Fretheim A, Nabudere H. Factors that influence the provision of intrapartum and postnatal care by skilled birth attendants in low- and middle-income countries: a qualitative evidence synthesis. Cochrane Database of Systematic Reviews 2017; 11(11): CD011558.

11 Hoope-Bender Pt, Bernis Ld, Campbell J, et al. Improvement of maternal and newborn health through midwifery. The Lancet 2014; 384(9949): 1226-35.

12 Lerberghe WV, Matthews Z, Achadi E, et al. Country experience with strengthening of health systems and deployment of midwives in countries with high maternal mortality. The Lancet 2014; 384(9949): 1215-25.

For peer review only



For peer review only - http://bmjopen.bmj.com/site/about/guidelines.xhtml

13 Adegoke A, Utz B, Msuya SE, Broek Nvd. Skilled Birth Attendants: Who is Who? A Descriptive Study of Definitions and Roles from Nine Sub Saharan African Countries. PLoS ONE 2012; 7(7): e40220.

14 Högberg U. The Decline in Maternal Mortality in Sweden: The Role of Community Midwifery. American Journal of Public Health 2004; 94(8): 1312-20.

15 Homer CSE, Friberg IK, Dias MAB, et al. The projected effect of scaling up midwifery. The Lancet 2014; 384(9948): 1146-57.

16 Heckman J, Robb R. Alternative methods for evaluating the impact of interventions: An overview. Journal of Econometrics 1985; 30(1-2): 239-67.

[dataset] 17 National Population Commission - NPC/Nigeria, ICF International. Nigeria Demographic and Health Survey 2013. Abuja, Nigeria: NPC/Nigeria and ICF International, 2014. https://dhsprogram.com/publications/publication-fr293-dhs-final-reports.cfm (access Feb 11, 2021)

[dataset] 18 Federal Government of Nigeria. 2014. Nigeria MDG Information System Nigeria Facility Inventory dataset. https://myf.github.io/nmis/about (accessed Feb 11, 2021).

19 Okwaraji YB, Edmond KM. Proximity to health services and child survival in low- and middle-income countries: a systematic review and meta-analysis. BMJ Open 2012; 2(4): e001196.

20. To access the Appendix, click on the Appendix link in the box to the right of the article online

21 ISRCTN. Improving maternal and child health care in Nigeria: an assessment of the maternal and child care component of the Subsidy Reinvestment & Empowerment Programme (SURE-P MCH). http://www.who.int/trialsearch/Trial2aspx?TrialID=ISRCTN98056858 2013; (1): 1-8.

22 Okwaraji YB, Edmond KM. Proximity to health services and child survival in low- and middle-income countries: a systematic review and meta-analysis. BMJ Open 2012; 2(4): e001196.

23 Okeke, E. N., & Abubakar, I. S. Healthcare at the beginning of life and child survival: Evidence from a cash transfer experiment in Nigeria. Journal of Development Economics 2020: 143:102426.

24 Neelsen S, Walque D de, Friedman J, *et al.* Financial Incentives to Increase Utilization of Reproductive, Maternal, and Child Health Services in Low- and Middle-Income Countries: A Systematic Review and Meta-Analysis. *Policy Res Work Pap* Published Online First: 2021. doi:10.1596/1813-9450-9793

25 Croke K, Mengistu AT, O'Connell SD, *et al.* The impact of a health facility construction campaign on health service utilisation and outcomes: analysis of spatially linked survey and facility location data in Ethiopia. *Bmj Global Heal* 2020;**5**:e002430. doi:10.1136/bmjgh-2020-002430

For peer review only

26 Burgert CR, Colston J, Roy T, Zachary B. Geographic displacement procedure and georeferenced data release policy for the Demographic and Health Surveys [Internet]. Calverton, Maryland, USA: ICF International; 2013. (DHS Spatial Analysis Reports No. 7). Available from: http://dhsprogram.com/pubs/pdf/SAR7/SAR7.pdf

For peer review only

Table 1: Summary of outcomes, characteristics of mothers and households, by study area, prior to the intervention

| | Total | | Intervention | | Comparison | | Difference | |
|---|---|---|---|---|---|---|---|---|
| Outcomes | N | % | N | % | N | % | Perc. Pt | p-value |
| Delivery assistance: doctor/nurse/midwife | 19,599 | 0.43 | 891 | 0.70 | 18,708 | 0.42 | 0.305 | 0.000 |
| Delivery in any facility | 19,671 | 0.44 | 891 | 0.72 | 18,780 | 0.43 | 0.317 | 0.000 |
| >=4 times of antenatal visits during pregnancy | 11,533 | 0.62 | 511 | 0.85 | 11,022 | 0.61 | 0.244 | 0.000 |
| | Total | | Intervention | | Comparison | | Difference | |
| Covariates | N | % | N | % | N | % | Perc. Pt | P-value |
| maternal age | 21,755 | 27.62 | 975 | 28.01 | 20,780 | 27.60 | 0.748 | 0.106 |
| parity at delivery | 21,755 | 2.84 | 975 | 2.24 | 20,780 | 2.87 | -0.613 | 0.000 |
| covered by health insurance | 14,074 | 0.02 | 648 | 0.03 | 13,426 | 0.02 | 0.004 | 0.592 |
| currently working | 14,065 | 0.73 | 645 | 0.80 | 13,420 | 0.73 | 0.094 | 0.000 |
| husband/partner has at least primary school education | 13,594 | 0.71 | 613 | 0.90 | 12,981 | 0.70 | 0.206 | 0.000 |
| respondent has at least primary school education | 14,136 | 0.63 | 648 | 0.86 | 13,488 | 0.62 | 0.266 | 0.000 |
| currently married or in union | 14,136 | 0.93 | 648 | 0.91 | 13,488 | 0.93 | -0.025 | 0.026 |
| respondent is Muslim | 14,075 | 0.53 | 646 | 0.35 | 13,429 | 0.54 | -0.264 | 0.003 |
| respondent reads newspapers | 14,136 | 0.18 | 648 | 0.29 | 13,488 | 0.18 | 0.104 | 0.014 |
| respondent listens to radio | 14,136 | 0.66 | 648 | 0.75 | 13,488 | 0.65 | 0.078 | 0.042 |
| respondent watches TV | 14,136 | 0.55 | 648 | 0.77 | 13,488 | 0.54 | 0.229 | 0.001 |
| household has electricity | 12,519 | 0.59 | 608 | 0.81 | 11,911 | 0.58 | 0.268 | 0.000 |
| belongs to the poorest two quintiles | 12,634 | 0.32 | 613 | 0.09 | 12,021 | 0.33 | -0.241 | 0.000 |
| residence is urban | 12,634 | 0.47 | 613 | 0.71 | 12,021 | 0.45 | 0.285 | 0.001 |
| residence in North East | 12,634 | 0.15 | 613 | 0.05 | 12,021 | 0.15 | -0.097 | 0.000 |
| residence in North West | 12,634 | 0.26 | 613 | 0.05 | 12,021 | 0.27 | -0.260 | 0.000 |
| residence in South East | 12,634 | 0.11 | 613 | 0.22 | 12,021 | 0.10 | 0.190 | 0.107 |
| residence in South South | 12,634 | 0.15 | 613 | 0.15 | 12,021 | 0.15 | 0.035 | 0.631 |
| residence in South West | 12,634 | 0.17 | 613 | 0.36 | 12,021 | 0.16 | 0.171 | 0.078 |

note: (1) buffer=100m, and catchment area=2,500m
(2) for birth record level variables, baseline is defined as births prior to October 2012 when the SURE-P MCH programme started

Table 2: OLS regressions – effect of the intervention on institutional deliveries and use of antenatal care

Panel 1: Unadjusted (no controls)

| | DID Coef | | 95% Confidence Interval | | | P-value | N |
|---|---|---|---|---|---|---|---|
| Institutional delivery | | | | | | | |
| 2,000m | 0.075 | [ | -0.042 | 0.193 | ] | 0.209 | 19,475 |
| 2,500m | 0.072 | [ | -0.032 | 0.175 | ] | 0.175 | 22,343 |
| 3,000m | 0.050 | [ | -0.047 | 0.146 | ] | 0.316 | 24,524 |
| At least 4 times of ANC visits | | | | | | | |
| 2,000m | 0.048 | [ | -0.034 | 0.131 | ] | 0.254 | 12,279 |
| 2,500m | 0.059 | [ | -0.012 | 0.130 | ] | 0.103 | 14,095 |
| 3,000m | 0.032 | [ | -0.044 | 0.109 | ] | 0.406 | 15,473 |

Panel 2: Adjusted (with controls)

| | DID Coef | | 95% Confidence Interval | | | P-value | N |
|---|---|---|---|---|---|---|---|
| Institutional delivery | | | | | | | |
| 2,000m | 0.074 | [ | -0.000 | 0.148 | ] | 0.051 | 18,413 |
| 2,500m | 0.067 | [ | -0.005 | 0.138 | ] | 0.069 | 21,130 |
| 3,000m | 0.050 | [ | -0.008 | 0.107 | ] | 0.090 | 23,240 |
| At least 4 times of ANC visits | | | | | | | |
| 2,000m | 0.029 | [ | -0.037 | 0.095 | ] | 0.393 | 11,488 |
| 2,500m | 0.030 | [ | -0.028 | 0.087 | ] | 0.311 | 13,200 |
| 3,000m | 0.020 | [ | -0.029 | 0.069 | ] | 0.420 | 14,526 |

notes:

(1) Control variables include both mother's characteristics (maternal age, square of maternal age, birth order, mother's health insurance coverage, current working status, mother and husband/partner's education level, mother's marriage status, religion, and exposure to media) and household characteristics (access to electricity and asset quintiles).

(2) All standard errors are robust and clustered at DHS cluster level.

## Supplementary Materials

The Wald test of the common trend assumption between 2008 and 2012 was never rejected for our main outcomes: institutional delivery (P-value = 0.30) and attendance of at least four antenatal visits during delivery (P-value = 0.24). Figures 1 and 2 show the trends, with confidence intervals, for these two outcome variables in the intervention and comparison groups during the 4-year pre-programme period; they confirm that pre-programme trends are roughly parallel when accounting for sampling noise. The common trend test is, however, rejected for infant mortality (p-value=0.016) and skilled birth attendance (p-value=0.046), hence we refrain from reporting results for these outcomes.

Figure 1: More than four time of ANC visits during pregnancy (catchment area=2500m, buffer=100m)

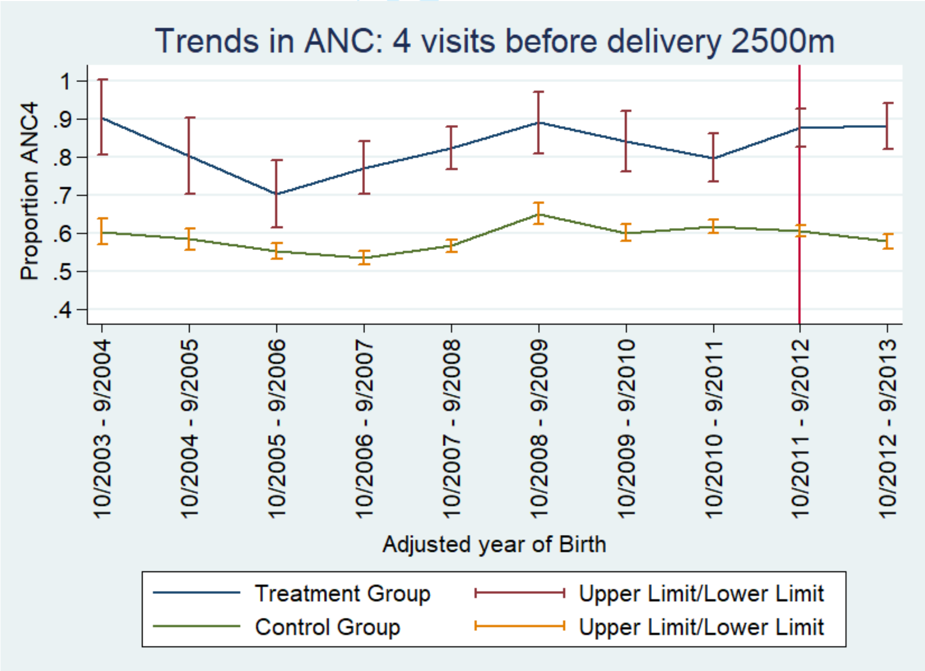

Figure 2: Skilled delivery (Catchment area=2500m, buffer=100m)

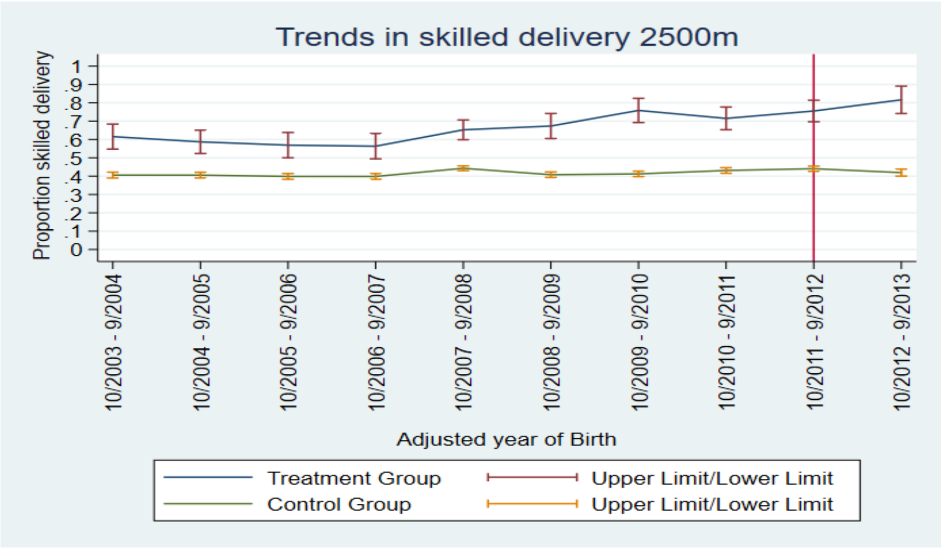


Figure 3: Institutional delivery (Catchment area=2500m, buffer=100m)

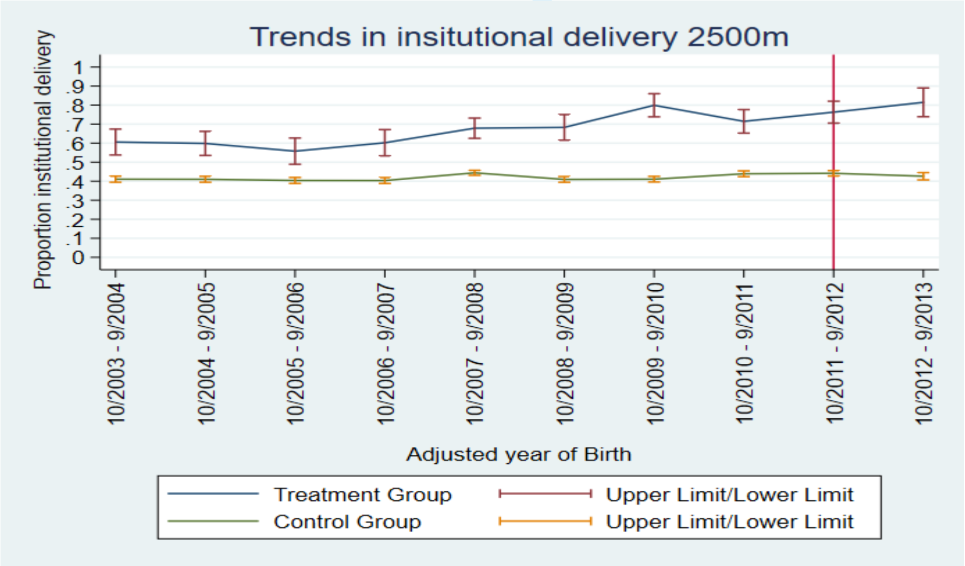

Table 1: Robustness check, estimates including data from the 2018 NDHS

***First year of SURE-P MCH : Oct 2012 - Oct 2013 (with controls)***

| | DID Coef | 95% Confidence Interval | | P-value |
|---|---|---|---|---|
| ***Institutional delivery*** | | | | |
| 2000m | **0.079** | 0.00 | 0.15 | **0.04** |
| 2500m | **0.068** | 0.00 | 0.14 | **0.06** |
| 3000m | **0.045** | -0.01 | 0.10 | **0.14** |
| *At least 4 times of ANC visits* | | | | |
| 2000m | **0.031** | -0.03 | 0.10 | **0.35** |
| 2500m | **0.032** | -0.02 | 0.09 | **0.26** |
| 3000m | **0.002** | -0.06 | 0.06 | **0.95** |

***Second (final) year of SURE-P MCH: Oct 2013 - Oct 2014 (with controls)***

| | DID Coef | 95% Confidence Interval | | P-value |
|---|---|---|---|---|
| ***Institutional delivery*** | | | | |
| 2000m | **-0.015** | -0.14 | 0.02 | **0.16** |
| 2500m | **0.019** | -0.10 | 0.07 | **0.67** |
| 3000m | **-0.015** | -0.09 | 0.06 | **0.68** |
| *At least 4 times of ANC visits* | | | | |
| 2000m | **-0.078** | -0.19 | 0.04 | **0.18** |
| 2500m | **0.052** | -0.15 | 0.04 | **0.29** |
| 3000m | **-0.049** | -0.13 | 0.04 | **0.26** |

ew only

STROBE Statement—Checklist of items that should be included in reports of ***cross-sectional studies***

| | Item No | Recommendation |
|---|---|---|
| **Title and abstract** | 1 | (*a*) Indicate the study's design with a commonly used term in the title or the abstract<br><br>The title now includes "a quasi-experimental study" |
| | | (*b*) Provide in the abstract an informative and balanced summary of what was done and what was found<br><br>We hope the abstract is informative and balanced |
| **Introduction** | | |
| Background/rationale | 2 | Explain the scientific background and rationale for the investigation being reported<br><br>Paragraphs 3-5 in the introduction provide background and rationale for the project |
| Objectives | 3 | State specific objectives, including any prespecified hypotheses<br><br>The final paragraph of the introduction states the aims of the project, we do not posit a hypothesis, however, we believe that it is likely that we would see an effect of increase midwives and facility upgrades on the use of health services. |
| **Methods** | | |
| Study design | 4 | Present key elements of study design early in the paper<br><br>We have included an "evaluation strategy" section in the Methods section of the paper. |
| Setting | 5 | Describe the setting, locations, and relevant dates, including periods of recruitment, exposure, follow-up, and data collection<br><br>We have provided context on the programme and study context in the Methods section of the paper. |
| Participants | 6 | (*a*) Give the eligibility criteria, and the sources and methods of selection of participants<br><br>We have provided a citation to the documentation for the Nigerian DHS, which provides more details on these elements of the survey. The survey is widely used and thus many readers will be familiar with the basics of this type of survey and as such we did not include a lot of details in the text. |
| Variables | 7 | Clearly define all outcomes, exposures, predictors, potential confounders, and effect modifiers. Give diagnostic criteria, if applicable<br><br>We provide a definition of our outcomes and predictors in the "data section" of the Methods. We list our control variables in the tables and the text. |
| Data sources/ measurement | 8* | For each variable of interest, give sources of data and details of methods of assessment (measurement). Describe comparability of assessment methods if there is more than one group<br><br>A citation is given to the data sources used. |



For peer review only - http://bmjopen.bmj.com/site/about/guidelines.xhtml

| | | |
|---|---|---|
| Bias | 9 | Describe any efforts to address potential sources of bias<br><br>We describe our empirical strategy, which has been developed to overcome potential sources of bias in our estimates. |
| Study size | 10 | Explain how the study size was arrived at<br><br>We discuss the sample of treated facilities, which dictates the study size. |
| Quantitative variables | 11 | Explain how quantitative variables were handled in the analyses. If applicable, describe which groupings were chosen and why<br><br>We describe in our “data sources” methods how we have handled our quantitative variables. |
| Statistical methods | 12 | (*a*) Describe all statistical methods, including those used to control for confounding |
| | | (*b*) Describe any methods used to examine subgroups and interactions |
| | | (*c*) Explain how missing data were addressed |
| | | (*d*) If applicable, describe analytical methods taking account of sampling strategy |
| | | (*e*) Describe any sensitivity analyses<br><br>We provide a discussion of these elements in our “data sources”. No steps were taken to address missing data. We have added a discussion of our robusteness checks. |
| **Results** | | |
| Participants | 13* | (a) Report numbers of individuals at each stage of study—eg numbers potentially eligible, examined for eligibility, confirmed eligible, included in the study, completing follow-up, and analysed |
| | | (b) Give reasons for non-participation at each stage |
| | | (c) Consider use of a flow diagram<br><br>Table 1 summarizes our sample |
| Descriptive data | 14* | (a) Give characteristics of study participants (eg demographic, clinical, social) and information on exposures and potential confounders |
| | | (b) Indicate number of participants with missing data for each variable of interest<br><br>Table 1 provides these data |
| Outcome data | 15* | Report numbers of outcome events or summary measures<br><br>Table 1 provides these data |
| Main results | 16 | (*a*) Give unadjusted estimates and, if applicable, confounder-adjusted estimates and their precision (eg, 95% confidence interval). Make clear which confounders were adjusted for and why they were included |
| | | (*b*) Report category boundaries when continuous variables were categorized |
| | | (*c*) If relevant, consider translating estimates of relative risk into absolute risk for a meaningful time period<br><br>Table 2 provides the results |
| Other analyses | 17 | Report other analyses done—eg analyses of subgroups and interactions, and |


For peer review only - http://bmjopen.bmj.com/site/about/guidelines.xhtml

| | | |
|---|---|---|
| | | sensitivity analyses<br><br>Table 2 has additional analysis in addition to our main results |
| **Discussion** | | |
| Key results | 18 | Summarise key results with reference to study objectives<br><br>First paragraph of the discuss summarizes the findings |
| Limitations | 19 | Discuss limitations of the study, taking into account sources of potential bias or imprecision. Discuss both direction and magnitude of any potential bias<br><br>There is a paragraph on the study limitations in the discussion |
| Interpretation | 20 | Give a cautious overall interpretation of results considering objectives, limitations, multiplicity of analyses, results from similar studies, and other relevant evidence<br><br>This is done in the discussion |
| Generalisability | 21 | Discuss the generalisability (external validity) of the study results<br><br>We have added a discussion of the comparability of our findings to existing literature to the discussion. |
| **Other information** | | |
| Funding | 22 | Give the source of funding and the role of the funders for the present study and, if applicable, for the original study on which the present article is based<br><br>This has been provided a statement in the role of the authors to state that the funding agency had not role in the preparation of the manuscript. |

*Give information separately for exposed and unexposed groups.

**Note:** An Explanation and Elaboration article discusses each checklist item and gives methodological background and published examples of transparent reporting. The STROBE checklist is best used in conjunction with this article (freely available on the Web sites of PLoS Medicine at http://www.plosmedicine.org/, Annals of Internal Medicine at http://www.annals.org/, and Epidemiology at http://www.epidem.com/). Information on the STROBE Initiative is available at www.strobe-statement.org.

For peer review only



For peer review only - http://bmjopen.bmj.com/site/about/guidelines.xhtml
